# Retraction: The crucial role of SRPK1 in IGF-1-induced EMT of human gastric cancer

**DOI:** 10.18632/oncotarget.28431

**Published:** 2024-08-14

**Authors:** Hong Wang, Chunlei Wang, Wenling Tian, Yanfen Yao

**Affiliations:** ^1^Department of General Surgery, Shandong Provincial Third Hospital, Jinan, Shandong, China; ^2^Thyroid Disease Prevention and Control Center, Shandong Provincial Institute of Endemic Disease Control, Jinan, Shandong, China; ^3^Department of Intensive Care Unit, Shandong Provincial Third Hospital, Jinan, Shandong, China

**This article has been retracted:** Figure 1C, presenting IHC staining for SRPK1 in gastric cancer tissues, contains a duplicate image of Figure 1C illustrating IHC staining for neogenin-1 in adjacent normal tissues of gastric cancer, from a previously published paper [1]. Figure 7B, presenting images of wound healing assay in gastric cancer cells, also contains a duplicate image from Figure 3A in a second previously published paper [2], studying epithelial ovarian carcinoma. And images of transwell assay In Figure 7C, are duplicates of images from Figure 6B and 6D in a third earlier published paper describing hepatocellular carcinoma study [3]. In light of these facts, the Editorial decision has been made to retract this paper. All authors agreed with the decision.

Original article: Oncotarget. 2017; 8:72157–72166. 72157-72166. https://doi.org/10.18632/oncotarget.20048
